# Conformational Flexibility of a Lipocalin Allergen (Mus m 1): Implications for Molecular Allergy Diagnostics

**DOI:** 10.3390/cimb47040234

**Published:** 2025-03-27

**Authors:** Federica Agosta, Thelma A. Pertinhez, Pietro Cozzini, Alberto Spisni, Elena Ferrari

**Affiliations:** 1 Molecular Modeling Laboratory, Food and Drug Department, University of Parma, 43121 Parma, Italy; federica.agosta@unipr.it (F.A.); pietro.cozzini@unipr.it (P.C.); 2 Laboratory of Biochemistry and Metabolomics, Department of Medicine and Surgery, University of Parma, 43125 Parma, Italy; elena.ferrari@unipr.it

**Keywords:** lipocalin allergen, Mus m 1, unpaired cysteine, conformational stability, conformational epitope, molecular allergology, molecular allergy diagnostics, component-resolved diagnosis

## Abstract

Mus m 1 lipocalin is the cause of mouse allergy in sensitized individuals. The production of a soluble, stable, and immunogenic isoform of Mus m 1 is essential for developing new diagnostic tools and immunotherapeutic protocols for treating allergic symptoms. To that end, using molecular dynamics (MD), we explored the impact of substitutions at positions 120 and 138 on the structure and dynamics of the allergic isoform Mus m 1.0102. HINT-based analysis of the MD trajectories, obtained for the mutants Y120F, Y120A, C138S, and C138A, allowed the assessment of the mutations’ impact on the network of intramolecular interactions, providing insights into the mechanisms underlying protein stability, dynamics, and allergenic reactivity. The C138A mutant revealed a reduction in the solvent-accessible surface area in the region of the mutated residue, of the radius of gyration, and of the α-helix displacement from the β-barrel, features that correlate with an increase in folding stability and a satisfactory allergenic potential. We consider C138A a good candidate to be exploited for diagnostic and vaccine purposes.

## 1. Introduction

Two decades of clinical research have revealed that numerous major human allergens belong to the lipocalin superfamily [1,2,3]. Several lipocalin-induced allergies, which are typically associated with respiratory pathologies, include hypersensitivity to pets, farm animals, and laboratory animals commonly encountered in indoor and in occupational settings [4,5,6]. The lipocalin allergens include the major mouse allergen Mus m 1, which is a complex of pheromone-binding isoforms called Major Urinary Proteins (MUPs): the major components of the mouse urinary protein fraction [7].

**Figure 1:**
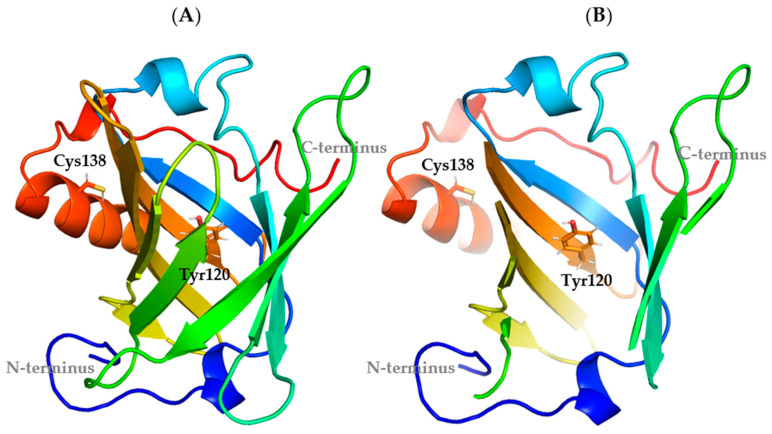
Crystal structure of a recombinant Mus m 1 allergen. (**A**) A cartoon representation of the Mus m 1.0102 structure (PDB: 1JV4). (**B**) A section of the β-barrel structure has been omitted to facilitate the visualization of the protein cavity. The residues selected for mutagenesis (in sticks) are Tyr120, at the bottom of the hydrophobic cavity, and Cys138, close to the C-terminal end of the α-helix.

Structural investigations of recombinant MUPs have revealed the presence of highly conserved motifs. Combining NMR and crystallography, we highlighted that the protein structure is characterized by: (i) an eight-stranded antiparallel β-barrel, hydrophobic inside and with only one entrance accessible to the solvent, thus allowing pheromone transfer; (ii) a C-terminal α-helix, which leans against the β-barrel wall, and (iii) three Cysteines, two of which are involved in a disulphide bond (Cys64-Cys157); the third unpaired one (Cys138) is located at the interface between the β-barrel and the α-helix (Figure 1A and Figure S1) [8,9], and it is responsible for protein aggregation [10].

Over the last decade, we have focused on the recombinant Mus m 1 component known as Mus m 1.0102, according to the WHO/IUIS Allergen Nomenclature, aiming at its structural optimization for clinical application. Having verified that the MUPs cavity has an overall flexibility that allows a variety of ligand binding modes [11], we employed a targeted mutagenesis approach to investigate the impact of Tyr120 and Cys138 (Figure 1B) on fold stability, unfold reversibility, and in vitro allergenicity (IgE reactivity) (Table 1).

The Tyrosine 120 side chain protrudes in the protein pocket, and it is involved in ligand binding through a hydrogen bond directly or via a water molecule [11]. Substitution with Phenylalanine (Y120F) eliminates the hydrogen bonding ability while it retains the aromatic and hydrophobic ring; alternatively, substitution with Alanine (Y120A) radically reduces the side chain’s steric hindrance while still preserving the cavity’s hydrophobicity. For both the Y120A and Y120F mutants, near-UV circular dichroism (near-UV CD) and intrinsic fluorescence spectroscopy revealed significant conformational rearrangements of the aromatic side chains within the internal cavity (Table 1) [10,12]. In vitro analysis of their allergenicity demonstrated a reduced IgE binding capability, indicating a potential alteration in their conformational epitopes (Table 1) [12].

The unique free Cys138 residue is acknowledged to cause inter- or intramolecular thiol/disulfide exchange, potentially leading to protein misfolding, dimerization, or aggregation [13]. Cysteine 138 was substituted by the isosteric and more polar Serine (C138S) or by the small and hydrophobic Alanine (C138A). These Cys138 substitutions did not significantly perturb the protein secondary structure or the organization of the aromatic residues, as evidenced by CD spectroscopy (Table 1). However, a reduction in thermal stability was observed for the C138S mutant, as monitored by intrinsic fluorescence and dynamic light scattering (Table 1), suggesting alterations in the protein conformation. Those conformational alterations enhanced the IgE binding’s efficacy (Table 1) [13]. The C138A mutant exhibited the highest resistance to thermal unfolding, indicating an augmented structural stability (Table 1), while its in vitro allergenicity turned out to be comparable to Mus m 1.0102 (Table 1) [10,14]. Of note, both the Cys138 mutants acquired unfolding reversibility, thus proving the role of the free thiol group in disulphide bond scrambling and protein misfolding (Table 1) [10,13]. Previous studies indicated that alteration of the hydrophobic interactions between the α-helix motif and the β-barrel may be responsible for the protein’s tendency to aggregate [10,13], thus pointing to a strict correlation between the protein’s structural and dynamic features and its function.

In this work, we analyzed the protein structural stability by combining a molecular dynamics approach with HINT (Hydropathic INTeractions), a force field-based program able to quantify hydrophobic and polar interactions in terms of an energy score that sums all atom–atom interactions [15,16,17,18]. The HINT-based analysis of the MD trajectories allowed the assessment of the effects of the single-point mutations on the network of intramolecular interactions that, by driving conformational alterations, may explain changes in the protein behavior and allergenic reactivity. This in silico study is a step forward to highlight molecular optimizations of Mus m 1.0102 toward its use for diagnostic and immunotherapy applications.

## 2. Materials and Methods

### 2.1. Structure Preparation

The crystallographic structure of recombinant Mus m 1.0102 (rMUP) was retrieved from the Protein Data Bank (https://www.rcsb.org/ accessed on 28 March 2024) (PDB ID: 1JV4; Resolution = 1.75 Å) [9]. The native ligand and co-crystallized water molecules were removed. Four different mutations, Y120F, Y120A, C138A, and C138S, were introduced into the wild-type structure using the PyMOL mutagenesis module (The PyMOL Molecular Graphics System, Version 1.8, Schrödinger, LLC, Cambridge, MA, USA).

Molecular systems were prepared and minimized with GROMACS v.2021.4 (https://www.gromacs.org/), using the Amber force field (ff19SB) [19]. Protein structures were (1) solvated in a triclinic box (10 Å radius) using the TIP3P water model, (2) centered in the box, (3) salted to 0.15 M NaCl, and neutralized using a Monte Carlo placing method [20]. Further details on the systems’ preparation are available in Table S1. Energy minimization was performed using the steepest descendent minimization algorithm and stopped when the maximum force was less than 100 KJ/(mol nm).

### 2.2. Molecular Dynamics Simulations

Molecular dynamics (MD) simulation allows the analysis of time-dependent motions of protein structures. Geometric quantities calculated from an MD simulation trajectory include the root mean square deviation (RMSD) and the root mean square fluctuation (RMSF) of atomic positions. RMSD values quantify how much a protein structure deviates from a reference structure over time. This means that the stability of a structure increases as the deviations decrease. The RMSF of a residue measures its flexibility during a simulation and is computed as the square root of the variance of the fluctuation around the residue’s average position.

MD simulations of the native and mutant structures of Mus m 1.0102 were run with GROMACS v.2021.4, choosing the Amber force field (ff19SB). Protein structures were prepared as described in Section 2.1. Each protein was centered and fit onto the starting position to remove some drawbacks due to periodic boundary conditions.

The RMSD and RMSF of the Cα atoms were calculated using the gmx rms and gmx rmsf modules of GROMACS, respectively. To identify significant differences between the RMSF profiles of the wild-type protein and the mutants, we arbitrarily selected the residues with RMSF increments or decrements of a magnitude ≥ 0.7 Å.

The systems were pre-equilibrated to the desired temperature and pressure. An NVT (constant Number of particles, Volume and Temperature) equilibration was performed in three different steps, each with a ΔT of 100 K. Then, an NPT (constant Number of particles, Pressure and Temperature) equilibration was applied for 0.5 ns to keep the system at a constant pressure of 1.0 bar. During the simulations, the temperature was maintained at 300 K using a Langevin thermostat [21], while the pressure was set at 1 bar using a Monte Carlo barostat [22].

The total simulation run time was 250 ns for each system. The molecular dynamics simulation time was chosen based on a prior analysis of the wild-type protein and the Y120 mutants, showing that it could capture the different conformational behaviors induced by single-point mutations.

Hydrogen bond lengths were constrained using the M-SHAKE algorithm with an integration time step of 2 fs [23]. Long-range Coulomb interactions were handled using the particle mesh Ewald (PME) summation method [24]. A non-bonded cut-off distance of 9 Å was used.

Principal component analysis (PCA) was performed with GROMACS to visualize large-scale collective motions of our systems on the trajectories generated by simulations. The first two principal components (PC1, PC2) were selected to analyze their projection of trajectories during the simulations.

The gmx gyrate and gmx sasa modules of GROMACS were used to calculate the radius of gyration (Rg) and solvent-accessible surface area (SASA), respectively.

The gmx distance module of GROMACS (used for calculating the distance between pairs of Cα positions as a function of time) was employed to evaluate the α-helix distance from the β-barrel during the simulation time. The following residues of the β-strands involved in the helix/barrel interface were considered: His20-Ser26, Phe100-Lys109, and Glu112-Gly121.

### 2.3. Intramolecular HINT Force-Field

The HINT program has proven to be a reliable and sensitive method for assessing protein thermodynamic stability by estimating the energy contributions due to intramolecular interaction patterns [18,25,26,27]. HINT (Hydropathic INTeractions) is a computational program based on the parameter logP_o/w_, where P_o/w_ is the 1-octanol/water partition coefficient. The log P_o/w_-based scoring function has been conceived to quantify hydrophobic and polar interactions between or within molecules in a biological environment [15,16]. Protein partitioning, which refers to the assignment of an atomic hydropathic parameter to each atom, was carried out under neutral pH conditions, using the “Dictionary” option of HINT [15] and the “semi-essential” hydrogen treatment. This approach includes polar, unsaturated, and alpha to heteroatom hydrogens. The hybridized pi/lone pair directionality vector was chosen to optimize the direction of the approach of two interacting atoms.

The intramolecular HINT score (*B*) is calculated as the sum of the hydropathic interactions between all atom pairs using the following equations:

$$B=\sum \sum b_{ij}$$

$$b_{ij}=S_{i\;}\;a_{i}\;S_{j}\;a_{j}\;R_{ij}$$

where *b_ij_* is a micro-interaction constant representing the attraction/interaction between atoms *i* and *j*; *S_i_* and *S_j_* are the solvent-accessible surface area for atoms *i* and *j*; *a_i_* and *a_j_* are the hydrophobic atom constants for atoms *i* and *j*, and *R_ij_* is the functional distance behavior for the interaction between atoms *i* and *j*. Calculations were carried out using the minimum energy structure for each protein.

### 2.4. Computational Resources

The molecular dynamics simulations required 150 h (CPU time) per protein on a high-performance computing (HPC) cluster. The computational analysis was performed using the HPC facilities provided by the Centro di Calcolo di Ateneo of the University of Parma.

## 3. Results

### 3.1. Molecular Dynamics Simulations

**Figure 2:**
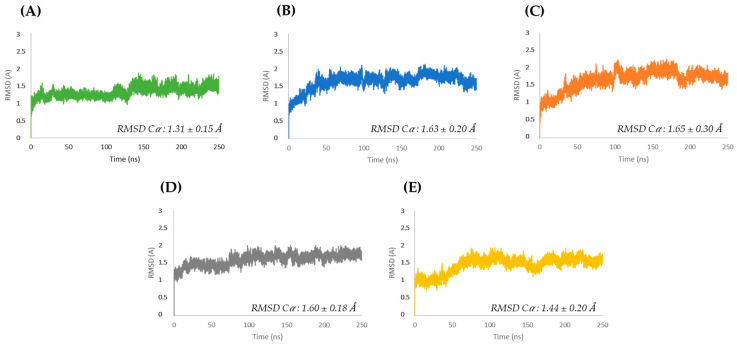
RMSD of Cα atoms as a function of computation time for Mus m 1.0102 (**A**) and its mutants: Y120F (**B**), Y120A (**C**), C138S (**D**), and C138A (**E**). Average RMSDs ± SD are given for all plots.

The MD calculations (Figure 2) describe the conformational dynamics of the native allergen Mus m 1.0102 and its mutants over 250 ns. Tests were initially carried out over 1200 ns, and it was found that after 250 ns, all the systems were reasonably stable. Thus, to save time and money, we chose to carry on all calculations over 250 ns. In this selected timescale, the average RMSD of the mutants was comparable to the resolution of the Mus m 1.0102 PDB structure (PDB ID: 1JV4 with R = 1.75 Å).

The Y120 mutants reveal a progressive and similar increase in RMSD, stabilizing at higher values than the other proteins (Figure 2B,C). In addition, at equilibration, the Y120A mutant (Figure 2C) exhibits larger RMSD oscillations than the Y120F mutant (Figure 2B). The wild-type and the C138S mutant exhibit a quick initial increase in RMSD, reaching equilibration after a transition at approximately 130 and 75 ns, respectively (Figure 2A,D). The C138A mutant achieves stability after a sigmoidal increase in RMSD during the first 75 ns (Figure 2E).

Root mean square fluctuation (RMSF) vs. residue plots identify the amino acids that contribute the most to protein flexibility. Except for specific residues in the terminal regions, the peaks of the conformational dynamics of Mus m 1.0102 correspond to the protein’s loops (L2, L3, L4, L5, L7), which are mainly exposed to the solvent (Figure S2A, top). The residues with an RMSF value of at least 2.5 Å are highlighted in the cartoon representation in Figure S2B.

**Figure 3:**
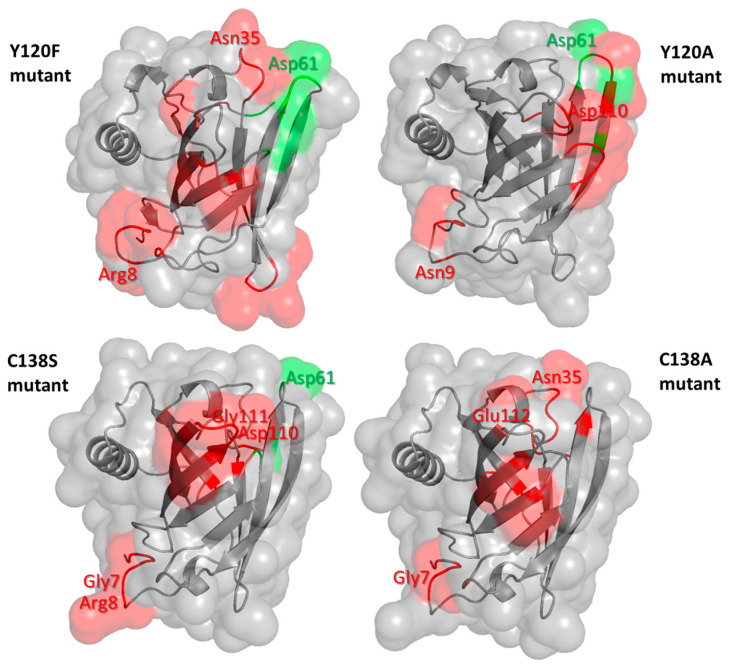
Regions of Y120F, Y120A, C138S, and C138A mutants with significantly altered flexibility compared to the wild-type protein. Residues (backbone and surface) showing a significant increase or decrease in flexibility (≥0.7 Å) are shown in red and green, respectively; the labelled residues have been predicted to be part of conformational epitopes of the wild-type protein by Discotope 2.0 server [29].

Similar RMSF profiles were obtained for the mutants (Figure S2A, bottom). Compared to the wild-type protein, the most significant RMSF alterations primarily involve the loop regions at the entrance and bottom of the protein pocket (Figure 3). Of note, some of these regions include residues predicted to be part of the conformational epitopes of Mus m 1.0102 [28], which may be involved in specific IgE binding in allergic subjects.

Principal component analysis (PCA) was used to evaluate the overall motion of the proteins by projecting each trajectory into a 2D essential subspace constructed from the first two principal components, PC1 and PC2 (Figure S3). The Mus m 1.0102 structure (Figure S3A) shows collective motions occupying the essential space between −2.59 and 2.88 nm on PC1 and between −1.57 and 2.86 nm on PC2, which indicates a rather compact structure. Despite a certain degree of overlap with the wild-type, the motions of the Y120F, Y120A, and C138S mutants cover different and larger areas of the PCA plot (Figure S3F,G), which correlates with their increased structural flexibility.

**Figure 4:**
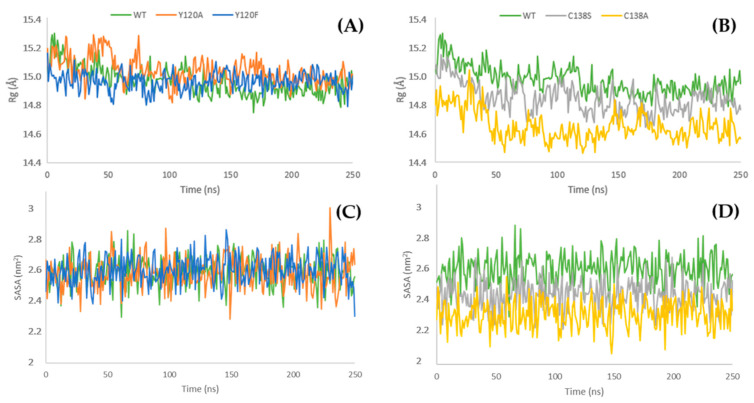
The radius of gyration (Rg) and solvent-accessible surface area (SASA) of Mus m 1.0102 and its mutants. (**A**) Rg plot of Mus m 1.0102 and its mutants Y120F and Y120A and (**C**) SASA plot of their residue 120. (**B**) Rg plot of Mus m 1.0102 and its mutants C138S and C138A and (**D**) SASA plot of their residue 138. The average Rg values calculated over the final 100 ns are: Mus m 1.0102, 14.90 ± 0.05 Å; Y120A mutant, 15.00 ± 0.06 Å; Y120F mutant, 14.96 ± 0.05 Å; C138S mutant, 14.80 ± 0.07 Å; C138A mutant, 14.63 ± 0.07 Å.

The analysis of the fluctuation of the radius of gyration (Rg) during MD calculations integrates the PCA data. In the final 100 ns, the Rg plots of the wild-type protein and the Y120F and Y120A mutants are quite similar (Figure 4A). Instead, the C138S and C138A mutants show an average Rg lower than the one of the wild-type. This finding suggests that these mutants possess a more densely packed structure than Mus m 1.0102 (Figure 4B).

A progressive reduction in solvent-accessible surface area (SASA) was observed for the residues S138 and A138 compared to the wild-type (Figure 4D). As expected for buried residues, the SASA of F120 and A120 remains unaltered (Figure 4C). In the last 100 ns, the Y120F, Y120A, and C138A structures reveal a minor increase in their global solvent exposure compared to the wild-type protein (Figure S4).

#### The Oscillatory Motion of the α-Helix Motif

**Figure 5:**
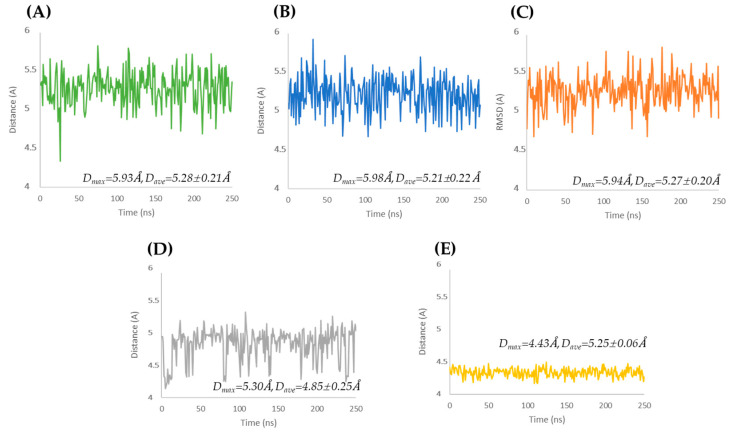
Conformational motions of the α-helix motif during the simulation time. The distance between the Cα atoms of the α-helix and the opposing β-strands is plotted against the simulation time. Mus m 1.0102 (**A**) and the mutants Y120F (**B**) and Y120A (**C**) are characterized by a significant α-helix displacement compared to the mutants C138S (**D**) and C138A (**E**). Each graph reports the maximum distance (D_max_) and the average distance (D_ave_) observed during the simulation.

Further analysis of the MD trajectories focused on estimating the distance of the α-helix motif from the β-strands. Figure 5A–C reports that the α-helix of Mus m 1.0102 and the Tyr120 mutants undergoes a comparable large oscillatory motion, moving up to 5.9 Å away from the β-barrel. Instead, the C138A mutant (Figure 5E) exhibits a markedly reduced α-helix displacement from the β-barrel (D_max_ = 4.4 Å, D_ave_ = 4.25 ± 0.06 Å) and a significantly reduced oscillation amplitude. The C138S mutant shows a sort of asymmetric oscillation (Figure 5D), with an average distance between the α-helix and the β-barrel of 4.85 ± 0.25 Å, a maximum distance of 5.3 Å, and a minimum distance of approximately 4.3 Å.

### 3.2. HINT-Based Analysis

HINT (Hydropathic INTeractions) is a fast and reliable energy scoring function able to estimate small energy differences when comparing intermolecular and intramolecular interaction patterns. The HINT score is a pure number obtained from experimental LogPo/w values based on the work of Abraham and Leo [30]. The novelty of this force field, developed and implemented by Kellogg and Abraham [16], is that, as HINT is based on empirical values, it is directly related to the free energy of binding (ΔG° = −RTLogP). HINT has been successfully employed to analyze and comprehend protein–ligand, protein–protein, and protein–DNA interaction energy [16,31]. Its relevance for intramolecular interactions has recently been validated for protein stability and flexibility analyses [18,25].

**Figure 6:**
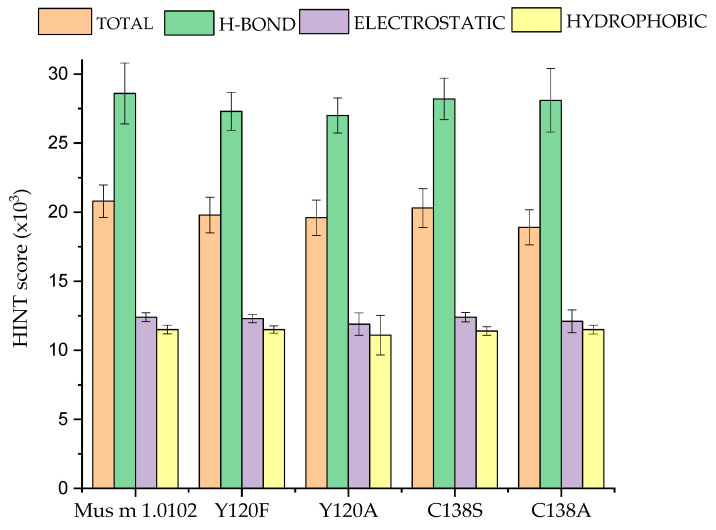
HINT scores of Mus m 1.0102 and its mutants. The bars (mean values ± SD) represent the total HINT score and the component scores resulting from the formation of hydrogen bonds, electrostatic interactions, and hydrophobic interactions.

In this study, a HINT-based analysis of the MD trajectories calculated for Mus m 1.0102 and its mutants provides clues on the energy contributions to their thermodynamic stability generated by the hydrogen bonds, hydrophobic interactions, and polar interactions occurring between all noncovalently bonded atom pairs. The protein total HINT score is the sum of favorable hydrogen bonds, electrostatic and hydrophobic interactions, and unfavorable repulsive Coulombic interactions and desolvation energies occurring within the protein itself. The minimum, maximum, and average total HINT scores for each protein are reported in Figure S5. The average energy contributions of the hydrogen bonds and electrostatic and hydrophobic interactions to the total average HINT score are reported in Figure 6.

The sensitivity of HINT to reveal small changes in the electrostatic and/or hydrophobic interaction energies at the atomic level can be appreciated by comparing the HINT scores obtained for the wild-type MUP and those of the mutants.

The average total HINT score of the mutants is comparable to that of the wild-type only for C138S, indicating that substituting a small polar residue with another one with similar characteristics does not significantly perturb the protein intramolecular interaction pattern. Moving to C138A, we observe a small reduction in the total average score. This is consistent with the apolar nature of Ala, and it is due primarily to a reduced contribution of the electrostatic interactions. When the Tyr120 residue is substituted by Phenylalanine, we note a clear reduction in the total average score, which is due mainly to the reduction in the hydrogen bond contribution, while the electrostatic and hydrophobic ones do not change. Indeed, removing the hydroxyl group hampers the possibility of forming hydrogen bonds, while the aromatic ring of the two amino acids provides the same capacity to establish electrostatic and hydrophobic interactions. Of note, these observations call for a comparison with the HINT scores obtained for C138A, where, as for the Tyr to Phe substitution, an H-bond donor is removed, thus hampering the possibility to form hydrogen bonds. Nonetheless, in this case, its hydrogen bond score does not appreciably change. This apparent discrepancy is interpreted as an indication that, in the native protein, the small Cysteine is unable to form any stable hydrogen bonds with other protein residues, whereas Tyr 120, thanks to its size and position in the protein architecture, can reach out to some residues.

**Figure 7:**
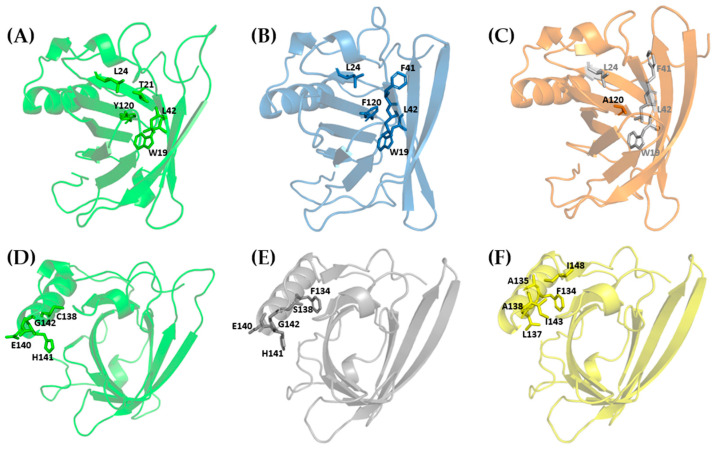
HINT-based interaction analysis of Mus m 1.0102 and its mutants. The structures display as colored sticks the residues exhibiting stronger interactions with the side chains of Y120 (**A**), F120 (**B**), A120 (**C**), C138 (**D**), S138 (**E**), and A138 (**F**). Residues shown as light grey sticks in (**C**) do not contribute to the interaction network of A120.

The HINT output in Table S2 reports the interactions involving the residues selected for mutation in Mus m 1.0102 and substituted in the mutants. The data enables a detailed analysis, at the atomic level, of the intramolecular interaction pattern present in Mus m 1.0102 and its mutants. Tyr120 of Mus m 1.0102 is involved in (i) a hydrogen bond with Thr21 via a water molecule; (ii) a network of hydrophobic interactions with Trp19, Leu24, and Leu42, and with Leu101 and Ala103, with minor hydrophobic scores (Figure 7A). Its substitution with Phenylalanine deletes the hydrogen bond while it preserves the hydrophobic network involving its aromatic ring (Figure 7B). The network is disrupted when Alanine replaces Tyrosine (Figure 7C). Alanine 120 can establish only feeble hydrophobic interactions with few residues inside the barrel, with an overall reduction in the hydrophobic contribution to the protein interactions network (Figure 6).

In Mus m 1.0102, Cys138 belongs to the α-helix motif and interacts via weak hydrogen bonds with Glu140, His141, and Gly142 (Figure 7D, Table S2). In other words, its interactions are mainly with residues of the α-helix motif, while they are very weak with the β-barrel. In the C138S mutant, those interactions are preserved. In addition, the hydroxyl group of S138 is involved in an H-bond interaction with the CO group of Phe134 (Figure 7E, Table S2), whose side chain projects toward the β-barrel. Overall, however, the wild-type interaction pattern is marginally affected by the mutation (Figure 6).

Conversely, a reduction in the HINT total average score is detected when Cys138 is substituted with Alanine for small variations in the hydrogen bond and electrostatic contributions (Figure 6). In the C138A mutant, in addition to the H-bonds involving Ala138 and stabilizing the α-helix motif, the Alanine side chain interacts with Phe134, Ala135, Leu137, Ile143, and Ile148, generating an extended hydrophobic patch on the α-helix surface (Figure 7F, Table S2). This peculiarity may explain the results reported in Figure 5, showing that in this mutant, the extension of fluctuation of the α-helix motif and its distance from the β-barrel is the smallest. It is conceivable to hypothesize that, to reduce the interaction with the polar solvent, the α-helix approaches the β-barrel surface until the attractive hydrophobic interactions reach a sort of equilibrium with the repulsive Coulombic ones.

## 4. Discussion

Allergy diagnosis is based on the reactivity of an allergen with specific IgE molecules present in the serum of allergic subjects. Natural allergen extracts are mixtures of substances with varying allergenic activity and allergen composition and have been used for in vivo and in vitro allergy diagnostics for decades, with poorly reproducible responses. Over time, many recombinant and natural single allergens have been isolated, purified, and characterized. These allergens are now accessible for component-resolved diagnostics (CRD) [32,33,34]. Indeed, the use of single-component preparations for diagnostics has opened the era of molecular allergology.

The demand for thorough allergen characterization and standardization has significantly increased. Developing an allergen preparation with fold stability and controlled allergenic potential is paramount for a personalized approach to the allergic patient for diagnostics and vaccine purposes. Unfortunately, the production of an allergen component that meets standardization requirements is time-consuming and often unsuccessful [35].

It is well recognized that exposure to mouse urinary proteins (MUPs) is a cause of allergic diseases among laboratory animal technicians and animal handlers, both in industry and academic research centers. Inhalation of MUPs produces IgE-mediated sensitization that originates symptoms of rhinitis, asthma, and, in some cases, dermatitis.

Due to its allergenic potential and structural resistance to temperature variation, the recombinant MUP, Mus m 1.0102, turned out to be a suitable diagnostic tool for Mus m 1 allergy [10,28]. However, its tendency to aggregate remains a limiting factor for any biomedical preparation [13].

Our current research focuses on structure mutagenesis and MD calculations to identify mutants of Mus m 1.0102 with suitable dynamic features and structural compactness of the helix/barrel interface. These two characteristics are essential to prevent its aggregation in time and preserve its allergenicity.

Based on evidence derived from previous studies [10,13,14], we analyzed the dynamic properties and compactness of Mus m 1.0102 mutants obtained by substituting Tyr120 or Cys138. Tyrosine 120, a conserved residue among MUPs, is part of the protein’s core structure (Figure 1) and is involved in ligand binding [10,28]. The unpaired Cys138 is located near the C-terminus of the α-helix (Figure 1), at the interface between the β-barrel and the α-helix, and it is known to cause inter- or intramolecular thiol/disulphide exchange, potentially interfering with unfolding/refolding processes [13].

The RMSD plots in Figure 2 illustrate that each mutant follows a specific trajectory toward equilibration that differs from the trajectory of the native Mus m 1.0102, reflecting the diverse initial structural constraints introduced by the point mutation. For all proteins, higher RMSF values correspond mainly to loop regions exposed to the solvent (Figure S2).

The PCA plots (Figure S3) highlight the increased flexibility of three out of four mutants compared to Mus m 1.0102. The fourth mutant, C138A, exhibits a plot that more closely resembles that of the native protein (Figure S3A,E), thus suggesting comparable structure stability.

Indeed, the Rg and SASA plots indicate that the C138A mutant is characterized by a more compact structure and by a reduced solvent exposure of the residue 138 (Figure 4B,D), which could be ascribed to its network of hydrophobic interactions with the neighboring residues, resulting in a reduction in the α-helix movement (Figure 5E) and a tightly packed structure. The observation of the distances between the α-helix and the β-barrel measured in the trajectory (Figure 5E) and the HINT-based analysis (Figure 6) confirm this model.

HINT assigns a lower total score to the Y120F and Y120A mutants than to the wild-type protein; this is caused primarily by the reduction in the hydrogen bonding score. However, in the case of Y120A, the reduction in the mean hydrophobic interaction score also plays an important role. In fact, according to the HINT-based interaction analysis, the hydrophobic residues that stabilize Tyr120 in the wild-type protein and Phe120 in the Y120F mutant (Figure 7A,B) do not interact with Ala120 in the Y120A mutant (Figure 7C). Based on these considerations, we would expect a destabilization of its core structure. Indeed, the Y120F and Y120A substitutions induce conformational alterations, resulting in a spatial rearrangement of the aromatic side chains that, for the Y120F mutant, leads to a reduced binding affinity for NPN (Table 1) [12] and for the Y120A mutant to tertiary structure alterations that we detected by near-UV and intrinsic fluorescence spectroscopy (Table 1) [12]. The data obtained for the C138S mutant indicate that the α-helix undergoes an asymmetric oscillation with an average distance of 4.85 Å from the β-barrel. Though further studies are required, we believe these peculiar oscillations (Figure 5D) and the conformational alterations of the backbone, which are close in space and sequence to the point mutation and have been evidenced by NMR chemical shift mapping [13], may be responsible for the decrease in thermal stability of the protein observed by intrinsic fluorescence and dynamic light scattering measurements (Table 1) [13]. As described in the Results Section, the α-helix of the C138A mutant exhibits quite different dynamic behavior; not only is its average distance from the β-barrel the smallest, 4.25 Å, but its fluctuation is markedly reduced, suggesting a more compact association between these structural elements. We hypothesized that the fluctuation of the α-helix would expose the hydrophobic residues of the helix/barrel interface to the solvent, thus favoring the observed aggregation propensity of Mus m 1.0102, particularly in response to temperature variations [13]. The restricted fluctuation of the α-helix observed in the C138A mutant can explain its enhanced thermal stability observed by intrinsic fluorescence and dynamic light scattering (Table 1) [10,14].

Overall, in the C138S and C138A mutants, the interactions of Tyr120 are consistent with those observed in the wild-type structure (Table S2), suggesting a good preservation of the core structure as highlighted previously by circular dichroism and intrinsic fluorescence spectroscopy. Moreover, both mutants exhibited a composition of secondary and tertiary structural elements like that of Mus m 1.0102 and, in the case of the C138A mutant, a comparable binding affinity for the ligand N-phenyl-1-naphthylamine, NPN (Table 1) [10,13].

In view of these observations, the C138A mutant represents a significant advance in the rational design of a Mus m 1 variant for biomedical applications in the field of molecular allergology. Its stability, enhanced compactness of the α-helix/β-barrel interface, and the resulting resistance to aggregation are valuable properties for its intended use in molecular diagnostics and immunotherapy.

Molecular determinants of allergenicity depend on the amino acid sequence of the allergen as well as on contributions from the allergen’s 3D structure and dynamics [36]. MD is currently employed to provide insights into the dynamics underlying allergenicity [37,38]. In food allergies, they have contributed to elucidating the effect of thermal processing on major epitopes of food allergens [39,40,41,42].

It has been postulated that structural flexibility allows allergens to interact with antibodies [37,38]. A recent MD study based on allergen–antibody complexes has demonstrated that epitopes exhibit less flexibility and plasticity, particularly when compared to antibody paratopes [43]. Other studies using different strategies confirmed an inverse relation between structural flexibility and antibody binding [44,45].

In a previous study, we employed Discotope 2.0 to predict the conformational epitopes of Mus m 1.0102. The predicted epitopes are epitope I, Glu1, Gly7, Arg8, Asn9, Asn11, Glu13; epitope II, Glu30, Asp34, Asn35, Asp61; and epitope III, Asp110, Gly111, Glu112 [28]. The MD analysis of Mus m 1.0102 shows that, among those residues, only Arg8 and Asp61 reveal exceptionally high RMSF values (≥4 Å), while the others range around 2 Å (Figure S2A), thus supporting the assumption that epitope residues may be less flexible and plastic.

The substitutions Y120F, Y120A, and C138S enhance the flexibility of some residues predicted to participate in Mus m 1.0102 epitopes while reducing only that of Asp61 (Figure 3). The resulting combinations of regions with altered flexibility may justify the reduced IgE binding capacity observed for the Y120F and Y120A mutants and the increased IgE reactivity of the C138S mutant [12,13]. In the C138A mutant, the ensemble of altered residue fluctuations and the increased compactness of the helix/barrel interface (Figure 3 and Figure 5E) correlate with an in vitro allergenicity comparable to that of Mus m 1.0102 [13]. Though the ensemble of these results provides useful hints, they also highlight the delicate and not yet fully understood balance of dynamic features that govern the reactivity of epitopes with specific antibodies. Indeed, the application of MD to lipocalin allergens that cross-react with Mus m 1 will help to develop and refine a mechanistic model for their reactivity to IgE antibodies.

## Figures and Tables

**Table 1:** Mus m 1.0102 and its mutants: a summary of previous findings from conformational and functional analyses.

Sample	Far-UV CD Spectroscopy	Near-UV CD Spectroscopy	Fluorescence Spectroscopy (Intensity)	NPN Ligand Binding Kd (±SE, nM)	Thermal Stability ^a^ T_m_, T_onset_ (°C)	Unfolding Reversibility ^b^	IgE Reactivity ^c^
Mus m 1.0102				25.0 ± 1.5	75.2, 62.5	No	
Y120F mutant	N/C	≠ ^d^	↑ ^e^	84.0 ± 24.0	-	-	↓ ^e^
Y120A mutant	N/C	≠≠	↑↑	-	-	-	↓↓
C138S mutant	N/C	N/C	N/C	-	71.9, 57.5	Yes	↑
C138A mutant	N/C	≠	N/C	33.8 ± 6.4	78.9, 70.0	Yes	N/C

Abbreviations: Far- and near-UV CD spectroscopy, circular dichroism spectroscopy providing information on secondary and tertiary structure, respectively; fluorescence spectroscopy, emission fluorescence of the protein’s unique Tryptophan (Trp19) providing information on tertiary structure; Kd, the dissociation constant of N-phenyl-1-naphthylamine (NPN), a probe for the ligand binding cavity of the protein; N/C, not significantly changed compared to Mus m 1.0102; T_m_, thermal transition midpoint measured by Trp19 fluorescence; T_onset_, temperature of unfolding onset measured by dynamic light scattering. ^a^ Thermal stability reflects the fold resistance to thermal stress and was assessed by fluorescence spectroscopy data as a function of temperature increase; ^b^ unfolding reversibility was assessed by CD and fluorescence spectroscopy upon reversing the temperature ramp; ^c^ IgE reactivity of proteins was assessed by an IgE-mediated degranulation assay using rat basophilic leukemia cells incubated with sera from mouse-allergic subjects (for IgE loading on specific receptors); ^d^ different spatial rearrangement of the aromatic side chains compared to Mus m 1.0102; ^e^ increased or decreased signal compared to Mus m 1.0102. Repeated symbols (e.g., ≠ ≠) indicate a more pronounced change than the simple symbol. Hyphen-minus corresponds to measurements not performed.

## Data Availability

Data are contained within the article and Supplementary Materials.

## Supplementary Materials

The following supporting information can be downloaded at https://www.mdpi.com/article/10.3390/cimb47040234/s1.
