# Supplementary material for: Conformational Flexibility of a Lipocalin Allergen (Mus m 1): Implications for Molecular Allergy Diagnostics
Source: Curr Issues Mol Biol. 2025 Mar 27;47(4):234. doi: 10.3390/cimb47040234 (PMC12026154; doi:10.3390/cimb47040234)
Supplement: Supplementary file 1 [file cimb-47-00234-s001.zip › cimb-3504959-supplementary.pdf]

Supplementary Materials

Conformational Flexibility of a Lipocalin Allergen (Mus m 1): Implications for Molecular Allergy Diagnostics

Federica Agosta [1], Thelma A. Pertinhez [2,*], Pietro Cozzini [1], Alberto Spisni [2,*] and Elena Ferrari [2]

[1] Molecular Modeling Laboratory, Food and Drug Department, University of Parma, 43121 Parma, Italy; federica.agosta@unipr.it (F.A.); pietro.cozzini@unipr.it (P.C.)
[2] Laboratory of Biochemistry and Metabolomics, Department of Medicine and Surgery, University of Parma, 43125 Parma, Italy; elena.ferrari@unipr.it
* Correspondence: thelma.pertinhez@unipr.it (T.A.P.); alberto.spisni@unipr.it (A.S.)

Table of contents

**Figure S1**. Protein chain features of Mus m 1.0102 (PDBsum, entry 1JV4).
**Figure S2.** RMSF analysis of Mus m 1.0102 and its mutants.
**Figure S3.** Principal Component Analysis (PCA) of the molecular dynamics' simulations.
**Figure S4.** Solvent accessible surface area (SASA) of Mus m 1.0102 and its mutants.
**Figure S5**. HINT score profiles of Mus m 1.0102 and its mutants.
**Table S1.** Systems preparation details for MD simulations.
**Table S2.** HINT table of intramolecular interactions.

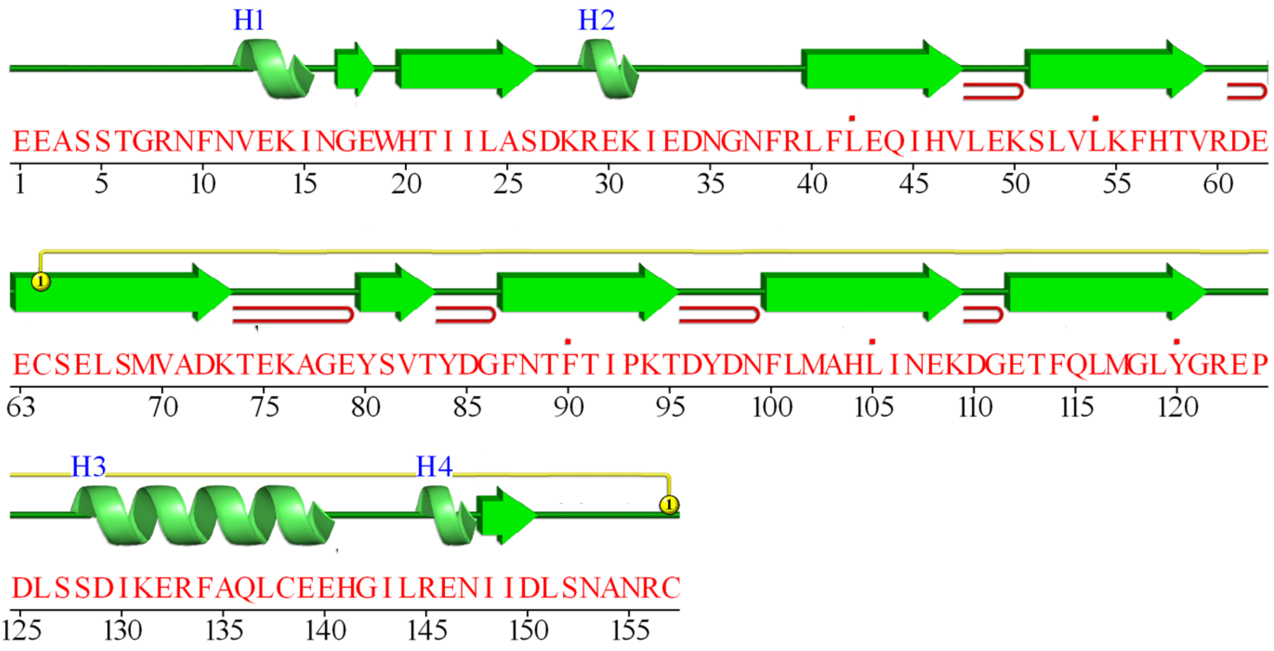

Figure S1. Protein chain features of Mus m 1.0102 (PDBsum, entry 1JV4). The β-strands and the helixes are shown as arrows and coils, respectively. The yellow line represents the disulphide bridge connecting Cys64 and Cys157. The curved pins are β-hairpins, and the red dots indicate the residues that interact with the native ligand 2-(sec-butyl)thiazole. The amino acid sequence does not include the N-terminal secretion signal.

(A)

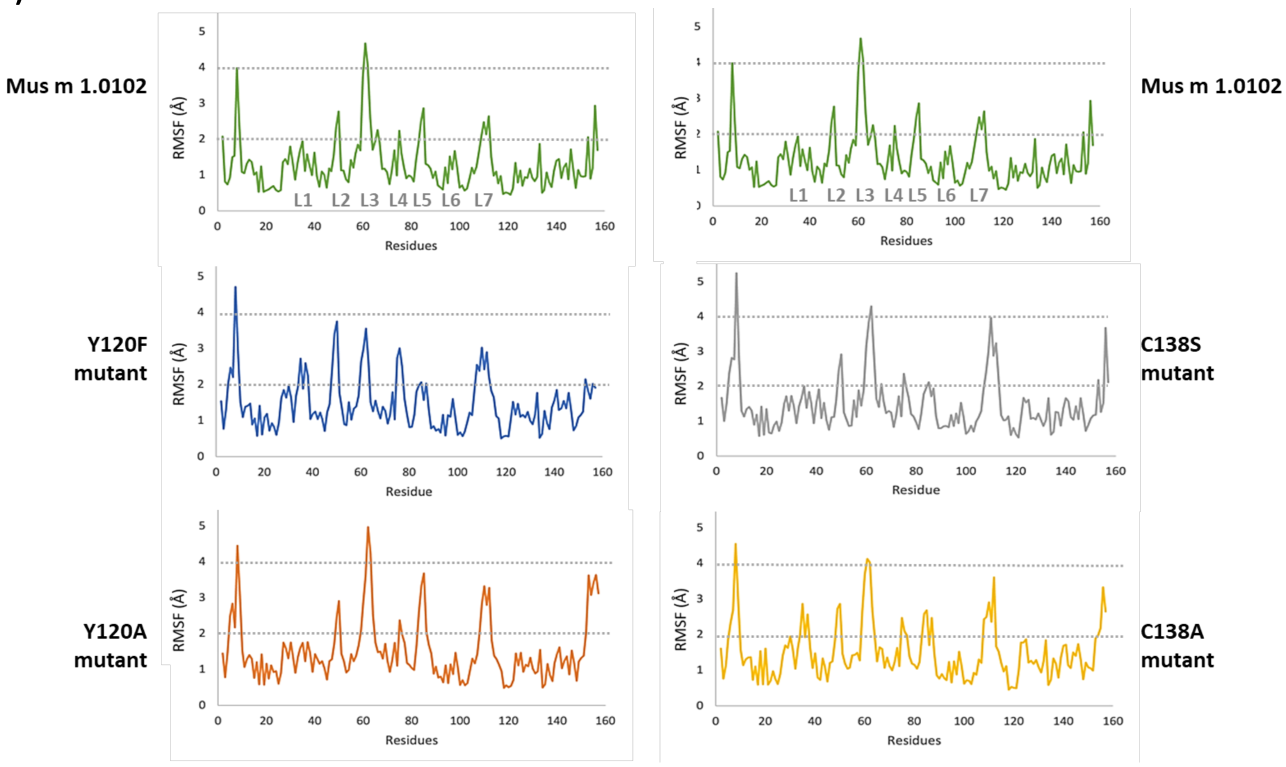

(B)

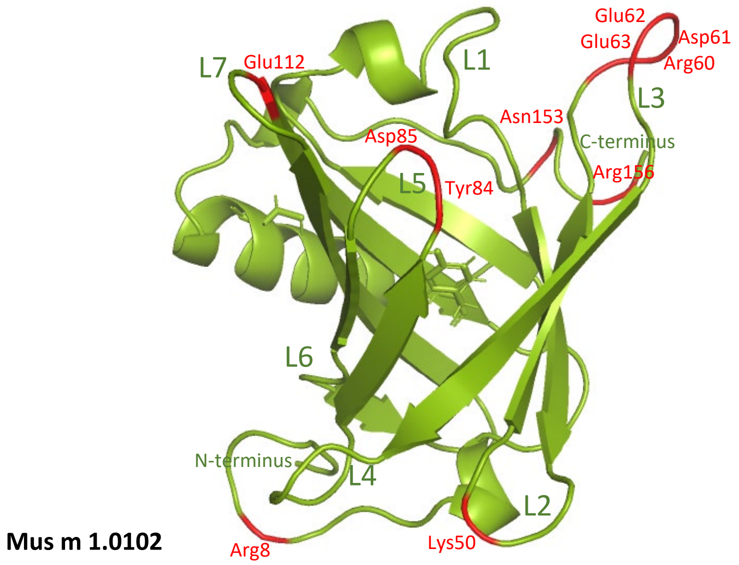

Figure S2. RMSF analysis of Mus m 1.0102 and its mutants (minimum energy conformations). (A) RMSF vs residue plot of Mus m 1.0102 (*top*) and its mutants: Y120F and Y120A (*left*); C138S and C138A (*right*). The RMSF peaks of the wild-type protein and the mutants correspond to residues mainly located within the protein loops (L1-7) and in the terminal regions of the structure. The grey dotted lines (Y=2 Å and Y=4 Å) were intended to facilitate the comparison of peak intensities. (B) The cartoon structure of Mus m 1.0102 (PDB code: 1JV4) is labelled and coloured in red according to the residues with the highest RMSF values (> 2.5 Å). Residues shown as sticks have been mutated to generate the mutant structures under study.

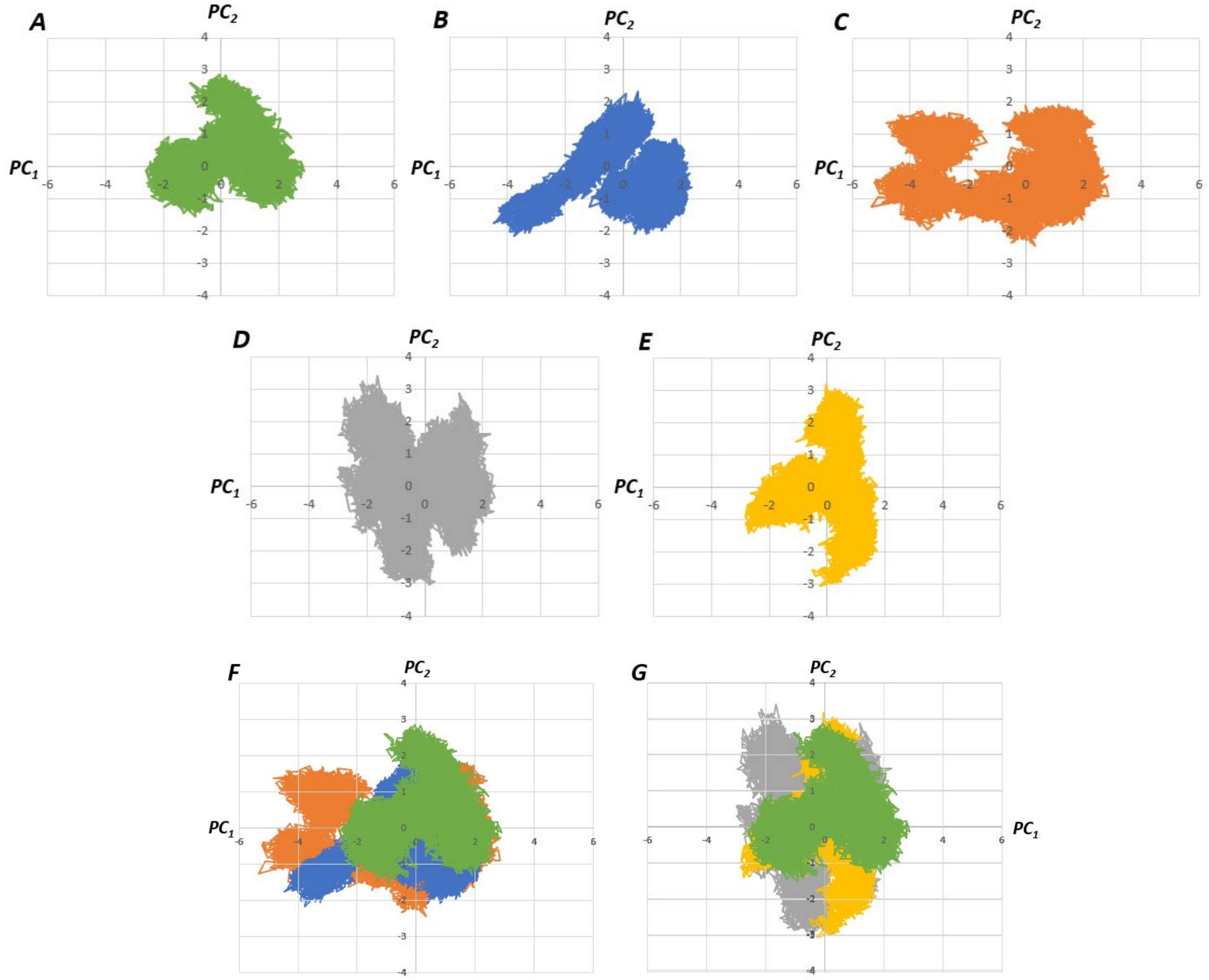

Figure S3. Principal Component Analysis (PCA) of the molecular dynamics' simulations. Projection of the motion for Mus m 1.0102 (A), Y120F (B), Y120A (C), C138S (D) and C138A (E) mutants. Plot superimposition of Mus m 1.0102 and Y120 mutants (F) or C138 mutants (G).

(A) (B)

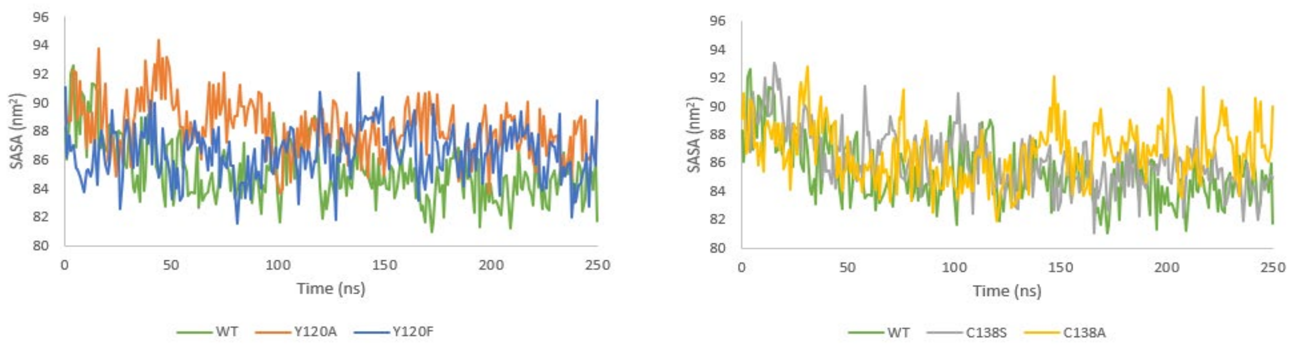

Figure S4. Solvent accessible surface area (SASA) of (A) Mus m 1.0102, Y120F and Y120A mutants, and of (B) Mus m 1.0102, C138S and C138A mutants.

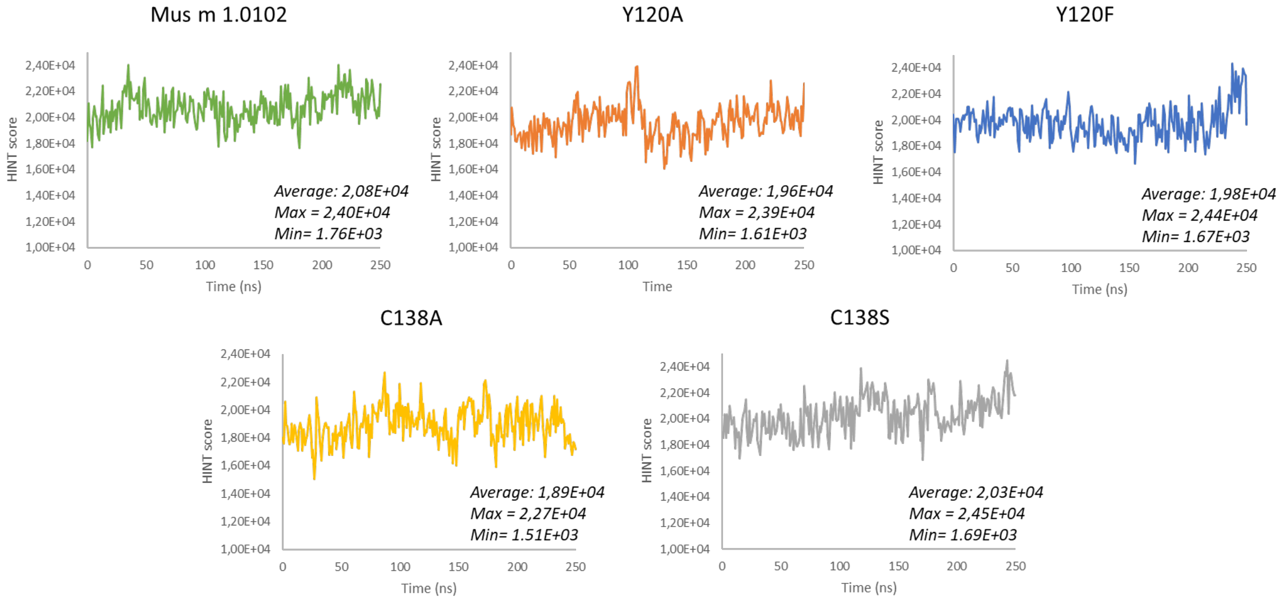

Figure S5. HINT score profiles of Mus m 1.0102 and its mutants. The minimum, maximum, and average HINT scores are indicated for each system. The HINT score (*total HINT score*) is calculated from the energy contributions due to hydrogen bonding and electrostatic and hydrophobic interactions.

Table S1. System preparation details for MD simulations.

System	Termini	Disulphide bond	Protonation State	Ions	Number of Water Molecules
Mus m1.0102	NTER, CSER	C64-C157	pH = 7.0	35 Na^+, 22 Cl^-	34008
Y120A	NTER, CSER	C64-C157	pH = 7.0	35 Na^+, 22 Cl^-	34014
Y120F	NTER, CSER	C64-C157	pH = 7.0	35 Na^+, 22 Cl^-	34008
C138A	NTER, CSER	C64-C157	pH = 7.0	35 Na^+, 22 Cl^-	34008
C138S	NTER, CSER	C64-C157	pH = 7.0	35 Na^+, 22 Cl^-	34017

Table S2. HINT table of the intramolecular interactions involving:

- Y120 and C138 in Mus m 1.0102
- F120 and C138 in Y120F mutant
- A120 and C138 in Y120A mutant
- S138 and Y120 in C138S mutant
- A138 and Y120 in C138A mutant.

The table includes the parameters used for calculation, such as the hydrophobic atom constant, the solvent-accessible surface area (SASA), and the distance between the interacting atoms. The final column describes the interaction type (hydrophobic, acid-based, acid-acid, base-base, hydrophobic-polar, or hydrogen bonding). Positive values represent favourable interactions for intramolecular stability.

Lines in italics indicate the hydrogen bonds that stabilize the α-helix and involve the residue at position 138.

Molecule: Mus m 1.0102

Monomer		Atom	Atom	Hydrophobic	SASA	Monomer		Atom	Atom	Hydrophobic	SASA	Distance		Interaction	
Type	Name	Name	Type	Atom Const.		Type	Name	Name	Type	Atom Const.		Angstroms	VDW	Score	Type
TRP	19	CB	C.3:2	0.489	30.00	**TYR**	**120**	CB	C.3:2	0.489	34.00	4.658	137.01	34	Hydrophobic
TRP	19	CZ3	C.ar	0.355	37.00	**TYR**	**120**	CB	C.3:2	0.489	34.00	4.476	131.64	21	Hydrophobic
TRP	19	CZ3	C.ar	0.355	37.00	**TYR**	**120**	O	O.2	-1.915	25.00	3.364	105.11	6	Hydrogen Bond
TRP	19	CE3	C.ar	0.355	19.00	**TYR**	**120**	CB	C.3:2	0.489	34.00	3.932	115.64	5	Hydrophobic
TRP	19	CE3	C.ar	0.355	19.00	**TYR**	**120**	O	O.2	-1.915	25.00	3.730	116.56	4	Hydrogen Bond
LEU	24	CD2	C.3:3	0.820	65.00	**TYR**	**120**	CE2	C.ar	0.355	35.00	4.981	146.51	31	Hydrophobic
LEU	42	CG	C.3:1	0.220	10.00	**TYR**	**120**	CD1	C.ar	0.355	22.00	4.158	122.31	16	Hydrophobic
LEU	42	CG	C.3:1	0.220	10.00	**TYR**	**120**	CE1	C.ar	0.355	35.00	4.171	122.68	22	Hydrophobic
LEU	42	CD1	C.3:3	0.820	66.00	**TYR**	**120**	CE1	C.ar	0.355	35.00	5.126	150.75	13	Hydrophobic

LEU	42	CD2	C.3:3	0.820	65.00	**TYR**	**120**	CB	C.3:2	0.489	34.00	4.610	135.59	10	Hydrophobic
LEU	42	CD2	C.3:3	0.820	65.00	**TYR**	**120**	CD1	C.ar	0.355	22.00	3.519	103.51	11	Hydrophobic
LEU	42	CD2	C.3:3	0.820	65.00	**TYR**	**120**	CE1	C.ar	0.355	35.00	3.730	109.69	9	Hydrophobic
PHE	100	N	N.am	-0.703	32.00	**TYR**	**120**	O	O.2	-1.915	25.00	4.514	147.98	6	Acid/Base
LEU	101	CD2	C.3:3	0.820	65.00	**TYR**	**120**	CB	C.3:2	0.489	34.00	4.395	129.27	12	Hydrophobic
LEU	101	CD2	C.3:3	0.820	65.00	**TYR**	**120**	CD2	C.ar	0.355	24.00	4.792	140.95	3	Hydrophobic
ALA	103	CB	C.3:3	0.810	75.00	**TYR**	**120**	CE2	C.ar	0.355	35.00	4.307	126.69	8	Hydrophobic
ALA	103	CB	C.3:3	0.810	75.00	**TYR**	**120**	CD2	C.ar	0.355	24.00	4.741	139.44	4	Hydrophobic
**CYS**	**138**	SG	S.3	-0.126	37.00	GLU	139	N	N.am	-0.519	31.00	4.737	141.41	-8	Acid/Acid
**CYS**	**138**	SG	S.3	-0.126	37.00	ILE	143	CB	C.3:3	0.810	75.00	5.465	156.14	-4	Hydroph./Polar
**CYS**	**138**	SG	S.3	-0.126	37.00	ILE	148	CB	C.3:3	0.810	75.00	5.683	162.38	-5	Hydroph./Polar
**CYS**	**138**	O	O.2	-1.915	25.00	GLU	140	N	N.am	-0.519	31.00	3.620	118.67	27	Hydrogen Bond
**CYS**	**138**	O	O.2	-1.915	25.00	HIS	141	N	N.am	-0.353	32.00	3.348	109.79	67	Hydrogen Bond
CYS	***138***	*O*	*O.2*	*-1.915*	*25.00*	*GLY*	*142*	*N*	*N.am*	*-0.703*	*35.00*	*2.962*	*97.11*	*101*	*Hydrogen Bond*

Molecule: Y120F mutant

TRP	19	CB	C.3:2	0.489	30.00	**PHE**	**120**	CB	C.3:2	0.489	29.00	4.133	121.55	51	Hydrophobic
TRP	19	CB	C.3:2	0.489	30.00	**PHE**	**120**	CD2	C.ar	0.355	18.00	4.530	133.25	27	Hydrophobic
ALA	22	CB	C.3:3	0.810	75.00	**PHE**	**120**	CA	C.3	0.173	8.00	4.591	135.02	3	Hydrophobic
ALA	22	CB	C.3:3	0.810	75.00	**PHE**	**120**	CB	C.3:2	0.489	29.00	5.950	175.00	2	Hydrophobic
LEU	24	CD1	C.3:3	0.820	66.00	**PHE**	**120**	CZ	C.ar	0.355	40.00	5.268	154.94	23	Hydrophobic
PHE	41	CB	C.3:2	0.489	29.00	**PHE**	**120**	CZ	C.ar	0.355	40.00	4.668	137.31	27	Hydrophobic
PHE	41	CB	C.3:2	0.489	29.00	**PHE**	**120**	CE2	C.ar	0.355	38.00	4.476	131.64	41	Hydrophobic
LEU	42	CB	C.3:2	0.520	23.00	**PHE**	**120**	CZ	C.ar	0.355	40.00	3.826	112.52	24	Hydrophobic
LEU	42	CB	C.3:2	0.520	23.00	**PHE**	**120**	CE2	C.ar	0.355	38.00	4.047	119.02	37	Hydrophobic
LEU	101	CB	C.3:2	0.520	23.00	**PHE**	**120**	CB	C.3:2	0.489	29.00	4.066	119.60	5	Hydrophobic
ALA	103	CB	C.3:3	0.810	75.00	**PHE**	**120**	CD1	C.ar	0.355	19.00	4.218	124.06	6	Hydrophobic

ALA	103	CB	C.3:3	0.810	75.00	**PHE**	**120**	CE1	C.ar	0.355	36.00	4.066	119.59	11	Hydrophobic
ALA	103	CB	C.3:3	0.810	75.00	**PHE**	**120**	CZ	C.ar	0.355	40.00	5.208	153.16	2	Hydrophobic
CYS	138	SG	S.3	-0.126	37.00	GLU	139	N	N.am	-0.519	31.00	4.797	143.19	-6	Acid/Acid
CYS	138	SG	S.3	-0.126	37.00	ILE	143	CB	C.3:3	0.810	75.00	4.864	138.98	-7	Hydroph./Polar
CYS	138	SG	S.3	-0.126	37.00	ILE	143	O	O.2	-1.915	28.00	5.328	161.47	2	Acid/Base
CYS	*138*	*O*	*O.2*	*-1.915*	*25.00*	*GLY*	*142*	*N*	*N.am*	*-0.703*	*35.00*	*3.087*	*101.22*	*90*	*Hydrogen Bond*
CYS	138	O	O.2	-1.915	25.00	GLU	140	N	N.am	-0.519	31.00	3.059	100.31	79	Hydrogen Bond
CYS	138	O	O.2	-1.915	25.00	HIS	141	N	N.am	-0.353	32.00	3.260	106.87	53	Hydrogen Bond

Molecule: Y120A mutant

TRP	19	CB	C.3:2	0.489	30.00	**ALA**	**120**	CB	C.3:3	0.810	75.00	4.278	125.84	14	Hydrophobic
TRP	19	O	O.2	-1.915	28.00	**ALA**	**120**	CB	C.3:3	0.810	75.00	5.944	185.75	-5	Hydroph./Polar
ALA	22	CB	C.3:3	0.810	75.00	**ALA**	**120**	CA	C.3	0.190	19.00	5.497	161.68	2	Hydrophobic
LEU	101	CB	C.3:2	0.520	23.00	**ALA**	**120**	CB	C.3:3	0.810	75.00	4.751	139.74	7	Hydrophobic
CYS	138	SG	S.3	-0.126	37.00	GLU	139	N	N.am	-0.519	31.00	4.821	143.90	-10	Acid/Acid
CYS	138	SG	S.3	-0.126	37.00	ILE	148	CB	C.3:3	0.810	75.00	5.490	156.86	-7	Hydroph./Polar
CYS	138	O	O.2	-1.915	25.00	GLU	140	N	N.am	-0.519	31.00	3.601	118.06	28	Hydrogen Bond
CYS	138	O	O.2	-1.915	25.00	HIS	141	N	N.am	-0.353	32.00	3.068	100.58	70	Hydrogen Bond
CYS	*138*	*O*	*O.2*	*-1.915*	*25.00*	*GLY*	*142*	*N*	*N.am*	*-0.703*	*35.00*	*3.065*	*100.49*	*59*	*Hydrogen Bond*

Molecule: C138S mutant

TRP	19	CB	C.3:2	0.489	30.00	TYR	120	CB	C.3:2	0.489	34.00	4.658	137.01	27	Hydrophobic
TRP	19	CZ3	C.ar	0.355	37.00	TYR	120	CB	C.3:2	0.489	34.00	4.476	131.64	13	Hydrophobic
LEU	24	CD1	C.3:3	0.820	66.00	TYR	120	CE1	C.ar	0.355	35.00	5.174	152.19	28	Hydrophobic
LEU	42	CB	C.3:2	0.520	23.00	TYR	120	CD1	C.ar	0.355	22.00	3.650	107.36	35	Hydrophobic
LEU	42	CB	C.3:2	0.520	23.00	TYR	120	CE1	C.ar	0.355	35.00	3.510	103.22	28	Hydrophobic
LEU	42	CD2	C.3:3	0.820	65.00	TYR	120	CB	C.3:2	0.489	34.00	4.505	132.50	11	Hydrophobic

LEU	42	CD2	C.3:3	0.820	65.00	TYR	120	CD1	C.ar	0.355	22.00	3.601	105.91	19	Hydrophobic
LEU	42	CD2	C.3:3	0.820	65.00	TYR	120	CE1	C.ar	0.355	35.00	4.029	118.49	17	Hydrophobic
LEU	101	CD2	C.3:3	0.820	65.00	TYR	120	CB	C.3:2	0.489	34.00	5.154	151.58	16	Hydrophobic
LEU	101	CD2	C.3:3	0.820	65.00	TYR	120	CE2	C.ar	0.355	35.00	4.951	145.61	13	Hydrophobic
LEU	101	CD2	C.3:3	0.820	65.00	TYR	120	CD2	C.ar	0.355	24.00	4.280	125.89	17	Hydrophobic
ALA	103	CB	C.3:3	0.810	75.00	TYR	120	CE2	C.ar	0.355	35.00	4.159	122.31	11	Hydrophobic
ALA	103	CB	C.3:3	0.810	75.00	TYR	120	CD2	C.ar	0.355	24.00	4.616	135.75	4	Hydrophobic
ALA	103	O	O.2	-1.915	28.00	TYR	120	OH	O.3	-0.440	40.00	5.614	187.14	11	Acid/Base
**SER**	**138**	O	O.2	-1.915	26.00	GLU	140	N	N.am	-0.519	31.00	3.484	114.22	30	Hydrogen Bond
**SER**	**138**	O	O.2	-1.915	26.00	HIS	141	N	N.am	-0.353	32.00	3.203	105.01	69	Hydrogen Bond
SER	***138***	*O*	*O.2*	*-1.915*	*26.00*	*GLY*	*142*	*N*	*N.am*	*-0.703*	*35.00*	*3.112*	*102.05*	*85*	*Hydrogen Bond*
PHE	*134*	*O*	*O.2*	*-1.915*	*26.00*	***SER***	***138***	*N*	*N.am*	*-0.224*	*32.00*	*2.950*	*96.72*	*109*	*Hydrogen Bond*
PHE	134	O	O.2	-1.915	26.00	**SER**	**138**	OG	O.3	-0.898	31.00	2.818	93.94	184	Hydrogen Bond

Molecule: C138A mutant

TRP	19	CB	C.3:2	0.489	30.00	TYR	120	CB	C.3:2	0.489	34.00	5.062	148.88	22	Hydrophobic
THR	21	CG2	C.3:3	0.810	67.00	TYR	120	CB	C.3:2	0.489	34.00	5.345	157.20	5	Hydrophobic
THR	21	CG2	C.3:3	0.810	67.00	TYR	120	CD1	C.ar	0.355	22.00	3.669	107.90	9	Hydrophobic
THR	21	CG2	C.3:3	0.810	67.00	TYR	120	CE1	C.ar	0.355	35.00	3.544	104.23	14	Hydrophobic
ALA	22	CB	C.3:3	0.810	75.00	TYR	120	CA	C.3	0.173	7.00	4.868	143.18	27	Hydrophobic
LEU	24	CD2	C.3:3	0.820	65.00	TYR	120	CE1	C.ar	0.355	35.00	4.869	143.21	31	Hydrophobic
LEU	42	CD1	C.3:3	0.820	66.00	TYR	120	CE1	C.ar	0.355	35.00	5.115	150.43	23	Hydrophobic
LEU	101	CG	C.3:1	0.220	10.00	TYR	120	CD2	C.ar	0.355	24.00	4.118	121.12	13	Hydrophobic
LEU	101	CD2	C.3:3	0.820	65.00	TYR	120	CB	C.3:2	0.489	34.00	4.572	134.47	20	Hydrophobic
LEU	101	CD2	C.3:3	0.820	65.00	TYR	120	CD2	C.ar	0.355	24.00	4.400	129.42	5	Hydrophobic
ALA	103	O	O.2	-1.915	28.00	TYR	120	OH	O.3	-0.440	40.00	4.920	163.99	23	Acid/Base
ALA	135	CA	C.3	0.190	19.00	**ALA**	**138**	CB	C.3:3	0.810	75.00	4.302	126.52	18	Hydrophobic

ALA	135	CB	C.3:3	0.810	75.00	**ALA**	**138**	CB	C.3:3	0.810	75.00	5.447	160.21	16	Hydrophobic
PHE	134	O	O.2	-1.915	26.00	**ALA**	**138**	CB	C.3:3	-0.898	31.00	2.818	93.94	14	Hydrophobic
LEU	137	CD2	C.3:3	0.820	65.00	**ALA**	**138**	CB	C.3:3	0.355	24.00	4.400	129.42	15	Hydrophobic
**ALA**	**138**	CA	C.3	0.190	19.00	GLU	139	CA	C.3	0.190	13.00	3.843	113.03	4	Hydrophobic
**ALA**	**138**	CA	C.3	0.190	19.00	GLU	140	N	N.am	-0.519	31.00	4.546	139.88	3	Hydroph./Polar
**ALA**	**138**	CA	C.3	0.190	19.00	HIS	141	N	N.am	-0.353	32.00	4.733	145.64	2	Hydroph./Polar
**ALA**	**138**	CA	C.3	0.190	19.00	HIS	141	CB	C.3:2	0.489	33.00	4.433	130.38	3	Hydrophobic
**ALA**	**138**	CA	C.3	0.190	19.00	ILE	143	CB	C.3:3	0.810	75.00	4.499	132.32	4	Hydrophobic
**ALA**	**138**	CB	C.3:3	0.810	75.00	ILE	143	CB	C.3:3	0.810	75.00	4.324	127.17	50	Hydrophobic
**ALA**	**138**	CB	C.3:3	0.810	75.00	ILE	148	CB	C.3:3	0.810	75.00	5.864	172.47	31	Hydrophobic
**ALA**	**138**	O	O.2	-1.915	28.00	GLU	140	N	N.am	-0.519	31.00	3.593	117.80	23	Hydrogen Bond
**ALA**	**138**	O	O.2	-1.915	28.00	HIS	141	N	N.am	-0.353	32.00	3.471	113.81	46	Hydrogen Bond
ALA	***138***	*O*	*O.2*	*-1.915*	*28.00*	*GLY*	*142*	*N*	*N.am*	*-0.703*	*35.00*	*3.205*	*105.07*	*54*	*Hydrogen Bond*
PHE	*134*	*O*	*O.2*	*-1.915*	*26.00*	***ALA***	***138***	*N*	*N.am*	*-0.703*	*32.00*	*3.155*	*103.44*	*111*	*Hydrogen Bond*
